# Erythropoietin and a hypoxia‐inducible factor prolyl hydroxylase inhibitor (HIF‐PHDi) lowers FGF23 in a model of chronic kidney disease (CKD)

**DOI:** 10.14814/phy2.14434

**Published:** 2020-05-31

**Authors:** Megan L. Noonan, Erica L. Clinkenbeard, Pu Ni, Elizabeth A. Swallow, Samantha P. Tippen, Rafiou Agoro, Matthew R. Allen, Kenneth E. White

**Affiliations:** ^1^ Department of Medical and Molecular Genetics Indiana University School of Medicine Indianapolis IN USA; ^2^ Department of Anatomy Cell Biology, and Physiology Indiana University School of Medicine Indianapolis IN USA; ^3^ Department of Medicine Division of Nephrology Indiana University School of Medicine Indianapolis IN USA

**Keywords:** CKD, EPO, FGF‐23, HIF‐PHDi, phosphate

## Abstract

Iron‐deficiency anemia is a potent stimulator of the phosphaturic hormone Fibroblast growth factor‐23 (FGF23). Anemia, elevated FGF23, and elevated serum phosphate are significant mortality risk factors for patients with chronic kidney disease (CKD). However, the contribution of anemia to overall circulating FGF23 levels in CKD is not understood. Our goal was to investigate the normalization of iron handling in a CKD model using the erythropoiesis stimulating agents (ESAs) Erythropoietin (EPO) and the hypoxia‐inducible factor prolyl hydroxylase inhibitor (HIF‐PHDi) FG‐4592, on the production of, and outcomes associated with, changes in bioactive, intact FGF23 (“iFGF23”). Our hypothesis was that rescuing the prevailing anemia in a model of CKD would reduce circulating FGF23. Wild‐type mice were fed an adenine‐containing diet to induce CKD, then injected with EPO or FG‐4592. The mice with CKD were anemic, and EPO improved red blood cell indices, whereas FG‐4592 increased serum EPO and bone marrow erythroferrone (Erfe), and decreased liver ferritin, bone morphogenic protein‐6 (Bmp‐6), and hepcidin mRNAs. In the mice with CKD, iFGF23 was markedly elevated in control mice but was attenuated by >70% after delivery of either ESA, with no changes in serum phosphate. ESA treatment also reduced renal fibrosis markers, as well as increased *Cyp27b1* and reduced *Cyp24a1* mRNA expression. Thus, improvement of iron utilization in a CKD model using EPO and a HIF‐PHDi significantly reduced iFGF23, demonstrating that anemia is a primary driver of FGF23, and that management of iron utilization in patients with CKD may translate to modifiable outcomes in mineral metabolism.

## Graphical abstract

Iron‐deficiency anemia is a potent stimulator of the phosphaturic hormone FGF23, and anemia, elevated FGF23, and serum phosphate are all significant risk factors for chronic kidney disease (CKD) patient death; however, the contribution of anemia to circulating FGF23 in CKD is not understood. We found that a CKD mouse model treated with EPO or a HIF‐PHDi to restore proper iron utilization had a 70% reduction of circulating iFGF23, normalization of vitamin D metabolic enzymes, and no exacerbation of hyperphosphatemia. In summary, our work demonstrated that the major portion of elevated iFGF23 in CKD is likely due to the associated anemia/iron deficiency, is therapeutically responsive to the control of iron utilization, and may thus provide modifiable patient benefit for mineral handling during CKD.

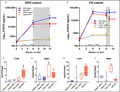

## 1 INTRODUCTION

Chronic kidney disease (CKD) is the most prevalent form of combined phosphate and iron handling defects. With declining renal mass, patients with CKD lose the ability to produce erythropoietin (EPO) and develop anemia due to low blood iron, inflammation, hemorrhage, EPO resistance, or relative EPO deficiency. Recombinant human EPO has become a central replacement therapy for a large number of patients with CKD, but despite improving anemia, patients with a higher targeted hemoglobin had more adverse events (Drueke et al., 2006). A new class of drugs, hypoxia‐inducible factor prolyl hydroxylase inhibitors (HIF‐PHDi) was developed to increase endogenous EPO while the kidney retains some functional capacity during CKD (Maxwell & Eckardt, 2016). HIF‐PHDi reduce the turnover of HIF1α and HIF2α transcription factors, with HIF2α driving kidney EPO production (Wiesener et al., 2003).

A key portion of the systemic response to reduce serum phosphate in CKD is to elevate bone‐produced bioactive, intact FGF23 (“iFGF23”). FGF23 acts through its co‐receptor αKlotho (KL) and fibroblast growth factor receptors (FGFRs) to decrease renal phosphate reabsorption (Kurosu et al., 2006). During CKD progression, with loss of renal mass and low KL expression, serum FGF23 steadily increases (Isakova, Wahl, et al., 2011). The marked increase in FGF23 during CKD goes beyond phosphate, as it was demonstrated in a mouse model of autosomal dominant hypophosphatemic rickets (ADHR) that iFGF23 was elevated during iron‐deficiency anemia in the genetic background of an *Fgf23* stabilizing mutation (Farrow et al., 2011). It was also shown in non‐dialysis patients with CKD that circulating FGF23 is associated with anemia (Nam et al., 2018). These findings suggest that there is crosstalk between phosphate and iron homeostasis via FGF23, a critical relationship since elevated FGF23 is associated with a > 6‐fold increased odds for CKD patient death (Isakova, Xie, et al., 2011).

The prevailing elevation of FGF23 in CKD is associated with marked endocrine disease. In this regard, increased FGF23 downregulates the renal anabolic vitamin D 1α‐hydroxylase (Cyp27b1) and upregulates the catabolic vitamin D 24‐hydroxylase (Cyp24a1) which lowers circulating active 1,25(OH)_2_ vitamin D (1,25D) (Shimada et al., 2001). The reduction in 1,25D results in hyperparathyroidism and subsequent metabolic bone disease, which can be severe. The modulation of FGF23 may improve patient outcomes, as an iFGF23 reduction of 30% in patients with CKD using a calcimimetic during the EVOLVE trial was associated with reducing both adverse vascular outcomes and all‐cause mortality (Moe et al., 2015). Thus reducing FGF23 in CKD to a level that does not induce hyperphosphatemia may provide critical ancillary benefit.

The role of iron utilization in driving FGF23 production appears somewhat paradoxical. Indeed, anemia clearly stimulates FGF23 mRNA production in bone in mice and humans with ADHR (Farrow et al., 2011; Imel et al., 2011). This relationship is underscored by the fact that oral iron supplementation to patients with ADHR normalized the elevated FGF23 and cured the prevailing hypophosphatemia (Imel et al., 2019). In contrast, certain types of intravenous iron preparations, such as those with carboxymaltose backbones, increase iFGF23, potentially through the interruption of normal FGF23 inactivation by intracellular protease cleavage (Wolf, Koch, & Bregman, 2013). Thus, both iron and anemia regulate the bioactive form of this hormone under specific conditions. We and others found that EPO and the HIF‐PHDi FG‐4592 raised FGF23 production in vitro and in vivo when given acutely (Clinkenbeard et al., 2017; David et al., 2016; Hanudel, Eisenga, et al., 2018); however, the effects of these therapeutic agents on circulating FGF23 concentrations during anemia, and its downstream effects on mineral metabolism, are unknown. Herein, we showed in an anemic CKD model that treatment with EPO and the HIF‐PHDi FG‐4592 improved iron utilization, and in parallel reduced iFGF23 and restored the balance of genes that control 1,25D production. Thus, careful management of anemia could provide important, modifiable outcomes for mineral metabolism in the CKD patient population.

## 2 MATERIALS AND METHODS

### 2.1 Animal studies

Animal studies were approved by, and performed according to the Institutional Animal Care and Use Committee (IACUC) of the Indiana University School of Medicine, and complied with the NIH guidelines for the use of animals in research. C57BL/6 female mice were purchased (Jackson Labs) and acclimated prior to study. Mice were euthanized by CO_2_ inhalation/cervical dislocation, and blood was collected by cardiac puncture for serum and plasma (collected in EDTA tubes). Where indicated, mice were facial vein bled for interim analysis, collecting less than 5% of the total blood volume to mitigate potential effects on the parameters tested (Diehl et al., 2001). Red blood cell indices were measured on whole blood from tail bleeds using an Element HT5 Veterinary Hematology Analyzer (Heska).

### 2.2 Rodent diets and ESA treatments

The adenine diet model was used to induce kidney failure in wild‐type mice, largely according to previous protocols (Clinkenbeard et al., 2019). Significant sex effects have been shown with the provision of adenine‐containing diets, therefore female mice were studied, as males rapidly succumb to the renal disease (Diwan, Small, Kauter, Gobe, & Brown, 2014). Mice were acclimated for 1 week and during this time fed normal rodent chow. At 8 weeks of age, mice were switched to a control casein‐based diet (0.9% phosphate and 0.6% calcium, TD.150303 Envigo), or a 0.2% “run‐in” adenine‐containing diet (TD.160020; Envigo) for 4 weeks. Mice were provided a 0.15% adenine “maintenance” diet (TD.150305; Envigo) after 4 weeks. Two different treatment protocols were employed to study the impact of the ESAs EPO and HIF‐PHDi. Mice receiving EPO (“EPO cohort”) were fed the adenine diet for 12 weeks total (4 weeks of 0.2% adenine, then 8 weeks of 0.15% adenine) to cause significant anemia. EPO injections (50 U/g, BioLegend) were given three times per week intraperitoneally (i.p.) starting at 6 weeks on the adenine diet. This dose was determined from previous reports of acute EPO injections in the adenine CKD mouse model (Hanudel, Eisenga, et al., 2018; Hanudel, Rappaport, et al., 2018). In contrast, mice receiving the HIF‐PHDi FG‐4592 (“FG cohort”) were tested on a shortened time course and were on the adenine diet for 9 weeks total (4 weeks of 0.2% adenine, then 5 weeks of 0.15% adenine) to ensure the mice with CKD were anemic but the kidneys were viable enough to produce endogenous EPO in response to a HIF‐PHDi dose comparable to other rodent studies (Beck, Henschel, Chou, Lin, & Del Balzo, 2017; Kabei et al., 2020; Seeley, Sternlicht, Klaus, Neff, & Liu, 2017). FG‐4592 injections (Roxadustat, SelleckChem; 70 mg/kg) were given i.p. three times over the last 5 days of the study. Diets and water were provided ad libitum.

### 2.3 Serum biochemical parameters

Routine serum biochemical parameters were determined using an automated COBAS MIRA Plus Chemistry Analyzer (Roche Diagnostics). Serum iFGF23 was assessed using a rodent‐specific commercial ELISA for bioactive, intact FGF23 (“iFGF23”; Quidel, Inc). Serum EPO was measured using the Mouse Epo Quantikine ELISA (R&D Systems).

### 2.4 RNA preparation and quantitative RT‐PCR (qPCR)

Kidney, liver, and bone marrow flushed from long bones were harvested and homogenized in 1 ml of Trizol reagent (Invitrogen/ThermoFisher Scientific) according to the manufacturer's protocol, then further purified using the RNeasy Kit (Qiagen, Inc). RNA samples were tested with intron‐spanning primers specific for mouse vitamin D 1α‐hydroxylase (*Cyp27b1*), vitamin D 24‐hydroxylase (*Cyp24a1*), type 1 collagen (*Col1a1*), type 3 collagen (*Col3a1*), erythroferrone (Erfe, *Fam132b*), ferritin (*Fth1*), bone morphogenetic protein‐6 (*Bmp6*), interleukin‐6 (*IL‐6*), and hepcidin (*Hamp*) mRNAs; *Gapdh* was used as an internal control for RT‐qPCR (Applied Biosystems/ThermoFisher Scientific). The TaqMan One‐Step RT‐PCR kit was used to perform the qPCR reactions under cycling conditions: 30 min 48°C, 10 min 95°C, followed by 40 cycles of 15 s 95°C and 1 min 60°C. The data were collected using a StepOne Plus system (Applied Biosystems/ThermoFisher Scientific). The expression levels of mRNAs were calculated relative to appropriate littermate diet controls, and data analyzed by the 2^−∆∆CT^ method (Livak & Schmittgen, 2001).

### 2.5 Statistical analysis

Data were analyzed by two‐way ANOVA, followed by a Tukey post hoc test or Student's *t* test (two‐tailed). Significance for all tests was set at *p* < .05. Data are represented as box‐and‐whisker plots with the middle line representing the median of the data, upper and lower quartiles within the boxes, and the whiskers as the minimum and maximum.

## 3 RESULTS

The primary use of the erythropoiesis stimulating agents (ESAs) EPO and HIF‐PHDi is to treat the anemia of CKD. Cohorts of wild‐type mice were provided an adenine‐containing diet to induce CKD as we previously described (Clinkenbeard et al., 2019), and were then treated with either recombinant human EPO (“EPO” cohort) or the HIF‐PHD inhibitor FG‐4592 (“FG” cohort). Mice with CKD in the EPO cohort treated with saline had significantly elevated creatinine and blood urea nitrogen (BUN) compared with control‐diet fed mice treated with saline, indicating renal disease induction (Table 1). Similar to patients with CKD, serum phosphorous was significantly elevated in the CKD‐saline group, and EPO treatment did not cause a significant difference to these levels (Table 1, *EPO cohort*). In the FG‐4592 cohort, BUN was elevated in mice with CKD compared with mice fed the casein diet (Table 1, *FG cohort*). There was no significant difference in serum creatinine, calcium, or phosphorous with either diet administration or drug treatment (Table 1, *FG cohort*). Analysis of red blood cell indices in the EPO cohort showed that the CKD‐saline mice were anemic with significantly lower RBC, HB, HCT, and MCV. EPO significantly increased these parameters in both casein control and CKD mice (Table 1, *EPO cohort*).

**TABLE 1:** Serum biochemical and red blood cell indices post‐treatment

	EPO cohort				FG cohort			
	Casein		CKD		Casein		CKD	
	Saline	EPO	Saline	EPO	Saline	FG	Saline	FG
BUN (mg/dl)	34.70 ± 0.53	35.85 ± 1.93	70.99 ± 6.14^ǂ^	62.91 ± 3.91^ǂ^	24.92 ± 0.99	56.93 ± 14.60	71.56 ± 3.87^ǂ^	70.00 ± 3.70
Creatinine (mg/dl)	0.44 ± 0.07	0.48 ± 0.06	0.71 ± 0.06^ǂ^	0.52 ± 0.03*	0.64 ± 0.13	0.72 ± 0.09	0.69 ± 0.07	0.59 ± 0.03
Alk Phos (U/L)	123.38 ± 8.1	187.71 ± 11.6**	134.25 ± 6.7	174.00 ± 11.2*	152.75 ± 13.2	75.00 ± 7.6**	168.75 ± 20.4	163.75 ± 21.4^ǂǂ^
Calcium (mg/dl)	8.52 ± 0.26	7.78 ± 1.16	9.00 ± 0.39	7.06 ± 0.69*	10.43 ± 0.63	10.40 ± 0.70	10.68 ± 0.60	10.33 ± 0.61
Phosphorous (mg/dl)	12.27 ± 0.31	13.34 ± 1.05	14.28 ± 0.76^ǂ^	14.30 ± 0.83	14.57 ± 0.69	17.03 ± 1.53	14.96 ± 1.07	15.53 ± 0.84
RBC (10^6^/μl)	10.64 ± 0.21	15.93 ± 0.48**	9.70 ± 0.31^ǂ^	17.18 ± 1.29**	9.75 ± 0.36	8.93 ± 0.46	8.57 ± 0.47	8.96 ± 0.56
HB (g/dl)	16.50 ± 0.35	24.18 ± 0.70**	13.10 ± 0.35^ǂǂ^	23.66 ± 0.58**	15.23 ± 0.56	13.9 ± 0.60	11.12 ± 0.64^ǂǂ^	11.73 ± 0.93
HCT (%)	47.72 ± 0.93	74.04 ± 1.84**	38.64 ± 0.96^ǂǂ^	78.62 ± 5.15**	45.17 ± 1.02	41.85 ± 1.55	34.62 ± 1.96^ǂǂ^	35.2 ± 2.49
MCV (fL)	44.86 ± 0.13	46.16 ± 0.58	39.86 ± 0.47^ǂǂ^	45.90 ± 0.84**	46.4 ± 0.70	46.85 ± 0.65	40.44 ± 0.77^ǂǂ^	39.2 ± 0.36
MCHC (g/dl)	34.62 ± 0.30	32.64 ± 0.29**	33.88 ± 0.23	30.4 ± 1.20**	33.33 ± 0.52	33.17 ± 0.15	32.12 ± 0.27^ǂǂ^	33.18 ± 0.36
Total iron (μmol/L)	29.55 ± 1.17	66.69 ± 5.74**	26.06 ± 1.73	69.70 ± 5.27**	36.4 ± 1.41	19.67 ± 2.24**	18.13 ± 3.34^ǂǂ^	13.63 ± 1.20

Note

Data presented as mean ± *SEM*.

Abbreviations: Alk phos, alkaline phosphatase; BUN, blood urea nitrogen; HB, hemoglobin; HCT, hematocrit; MCHC, mean corpuscular hemoglobin content; MCV, mean corpuscular volume; RBC, red blood cell count.

*n* = 3–8 mice/group; **p < .01 versus. treatment, same diet; ^ǂ^ *p* < 0.05, ^ǂǂ^ *p* < 0.01 versus. diet, same treatment via two‐way ANOVA with a Tukey post hoc test.

**FIGURE 1:**
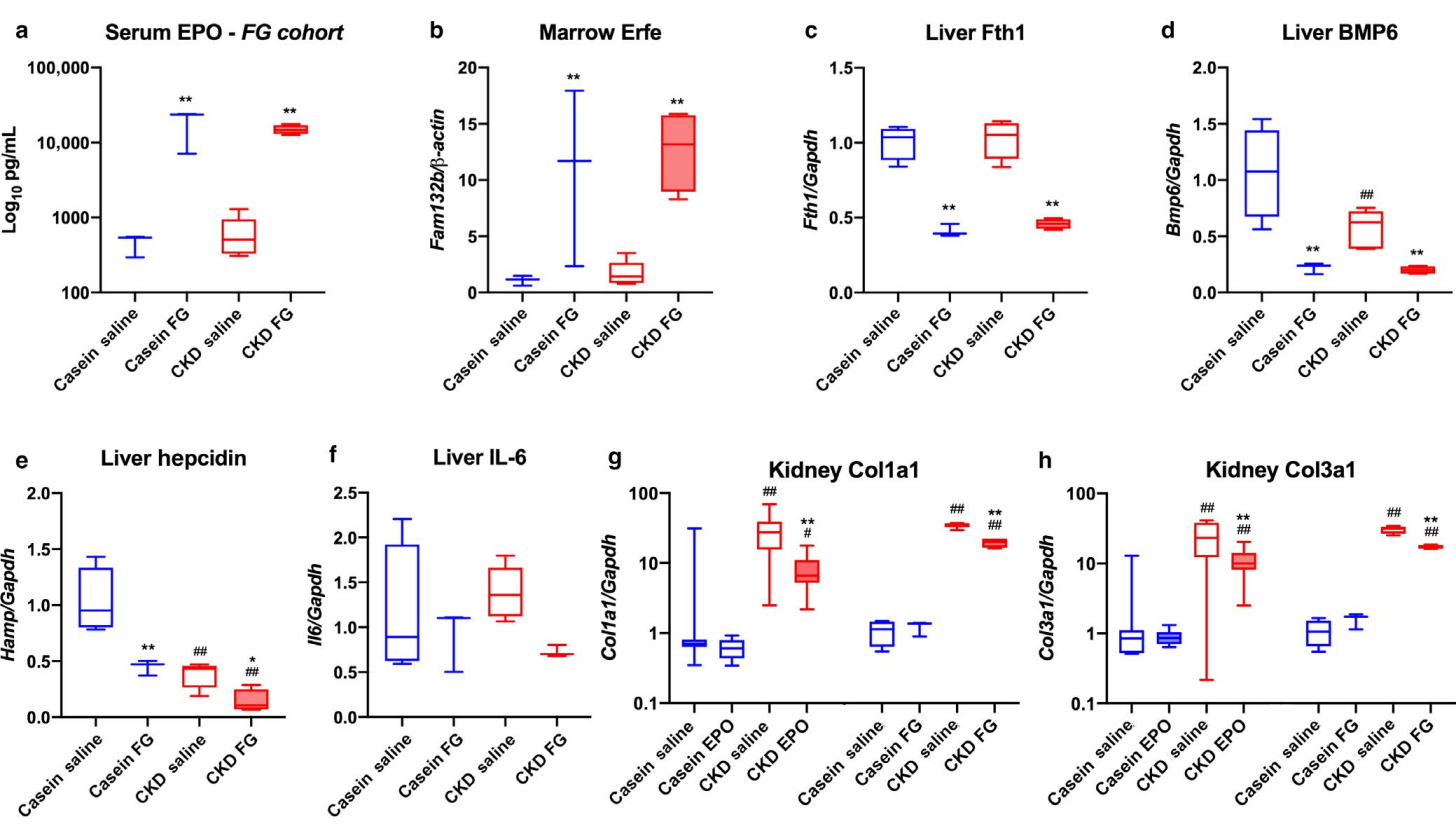
Iron utilization parameters and markers of renal fibrosis in CKD mice treated with ESAs. (a) Mice treated with FG‐4592 had markedly elevated serum EPO in both casein and CKD mice. (b) Bone marrow Erythroferrone (Erfe) expression was significantly elevated in both normal and CKD mice treated with FG‐4592. (c) Liver ferritin (Fth1) mRNA expression was reduced with FG‐4592 treatment in both casein and CKD groups. (d) Liver Bmp‐6 and (e) Hepcidin expression was decreased in both control and CKD mice with FG treatment, and was reduced in saline‐CKD mice compared to casein controls. (f) Liver IL‐6 trended toward being reduced with FG‐4592 treatment in normal and CKD mice. (g) Fibrosis marker Col1a1 was significantly elevated in CKD‐saline mice in both the EPO and FG cohorts; treatment with either EPO or FG‐4592 significantly reduced expression. (h) Similarly, Col3a1 was upregulated in CKD‐saline mice in both groups and was significantly reduced with ESA treatment (*n* = 3–8 mice per group; *p < .05; **p < .01 versus treatment, same diet; ^#^ *p* < .05; ^##^ *p* < .01 versus diet, same treatment via two‐way ANOVA with a Tukey post hoc test)

The FG‐4592 CKD cohort also had anemia prior to FG‐4592 treatment. FG‐4592 treatment rescued MCHC to control levels but did not significantly alter any other red blood cell parameters (Table 1, *FG cohort*). The EPO treatment significantly increased total serum iron in both control and CKD mice (Table 1, *EPO cohort*). In contrast, serum iron was reduced following acute FG‐4592 treatment in the control and CKD mice (Table 1, *FG cohort*) potentially due to increased iron utilization by stimulated erythropoiesis. We also observed that FG‐4592 significantly elevated serum EPO concentrations in both the diet‐control mice and the CKD group (Figure 1a). Additionally, FG‐4592 significantly elevated bone marrow erythroferrone (Erfe) mRNA expression, an EPO‐responsive hormone (Figure 1b). To address whether rapid changes in iron utilization occurred with FG‐4592 treatment, liver iron markers were assessed. Ferritin H (Fth1) mRNA expression was reduced in both control and CKD mice treated with FG‐4592 (Figure 1c). This reduction was associated with decreased liver Bmp‐6 (Figure 1d and hepcidin (Hamp) (Figure 1e) gene expression in both control and CKD groups, linking enhanced erythropoiesis with iron utilization. This was also associated with a trend toward reduced liver IL‐6 mRNA expression (Figure 1f). One of the pathogenic manifestations of CKD is the progressive and irreversible renal fibrosis inducing kidney damage (Duffield, 2014). We assessed whether EPO, previously described to alleviate renal fibrosis in CKD (Geng, Hu, & Lian, 2015), and FG‐4592, could impact markers associated with renal fibrosis, including type I (Col1a1) and type III collagen (Col3a1). Both Col1a1 (Figure 1g) and Col3a1 (Figure 1h) mRNAs were significantly elevated in the mice with CKD, and EPO and FG‐4592 reduced their expression.

**FIGURE 2:**
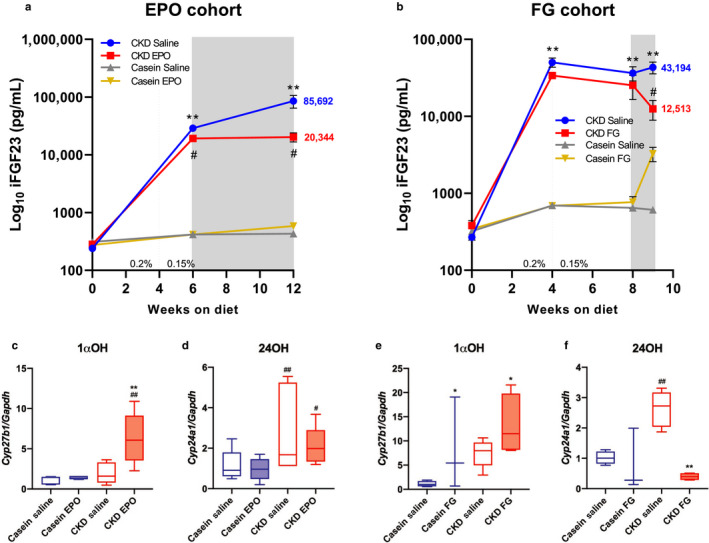
Effect of treatments on iFGF23 and 1,25D‐regulating enzyme expression. (a) Intact FGF23 (iFGF23) in the EPO cohort was markedly elevated in CKD‐saline mice compared to the casein control diet mice. EPO administration in the casein diet group had no effect on FGF23 compared to saline‐injected mice. Importantly, iFGF23 was lowered in CKD mice treated with EPO by over 70%. (b) In the FG cohort, the adenine diet induced iFGF23, which remained elevated in saline‐treated mice. FG‐4592 (“FG”) treatment in CKD mice significantly reduced iFGF23 levels by over 70% compared to saline controls. The dashed line in each graph indicates where diet was switched from 0.2% adenine to 0.15% adenine for the remainder of the study. The shaded area indicates the treatment window with either EPO or FG‐4592. (c) In the EPO‐treated cohort, renal Cyp27b1 mRNA remained unchanged in mice with CKD compared to casein controls, and EPO treatment significantly induced its expression. (d) Cyp24a1 mRNA was increased in mice with CKD compared with controls, and there was no significant difference in expression in the EPO‐CKD mice. (e) In the FG‐4592 treated cohort, Cyp27b1 mRNA was significantly elevated with FG‐4592 treatment in both diets with no difference between the diets. (f) Cyp24a1 expression was elevated in the mice with CKD, and this was reduced to control levels with FG‐4592 treatment (*n* = 3–8 mice per group; *p < .05;**p < .01 CKD Saline versus Casein‐Saline, ^#^ *p* < .05; ^##^ *p* < .01 CKD‐EPO/FG versus CKD Saline via two‐way ANOVA with a Tukey post hoc test or Student's *t* test where appropriate)

Similar to patients with CKD, blood iFGF23 rose progressively in the mice with CKD prior to ESA treatment, and continued to increase in the saline‐treated CKD mouse groups. The EPO administration in the control diet group had no effect on serum iFGF23. In stark contrast, iFGF23 was reduced by > 70% upon EPO treatment of the CKD cohort (Figure 2a). FG‐4592 treatment raised iFGF23 in mice fed the casein diet; however, there was no statistically significant difference when compared with saline‐treated mice receiving the same diet. In a manner similar to the CKD‐EPO‐treated mice, the mice with CKD in the FG‐4592 cohort had reduced levels of iFGF23 by over 70% at the end of the treatment period (Figure 2b). Therefore, the anemia of CKD is a potent stimulator of FGF23, as improvement of iron utilization via exogenous EPO administration, or endogenous EPO stimulation in mice treated with a HIF‐PHDi, markedly attenuated the increase in circulating iFGF23 concentrations.

Patients with CKD can have aberrant 1,25D production, due in part to the actions of elevated iFGF23 on the kidney (Mehrotra et al., 2009). To determine the downstream effects of lowered iFGF23 on 1,25D metabolism, kidney Cyp27b1 and Cyp24a1 gene expression was measured, and showed that although modestly elevated, there was no statistical difference in Cyp27b1 expression between casein‐saline and CKD‐saline groups; EPO treatment significantly elevated Cyp27b1 expression in the mice with CKD (Figure 2c). As expected from the prevailing elevated iFGF23, Cyp24a1 mRNA was significantly increased in the CKD group, whereas EPO treatment had a trend toward reduced expression in these mice (Figure 2d). Similarly, FG‐4592 treatment significantly increased Cyp27b1 in both the casein and CKD groups (Figure 2e). Further, the elevated Cyp24a1 was attenuated with FG‐4592 treatment (Figure 2f). Taken together, these data support the proposition that the changes that occur in 1,25D metabolism during CKD may be reversible by therapeutics targeting defective iron handling, potentially through lowering FGF23, but not to levels that could cause further increases in serum phosphate.

## 4 DISCUSSION

Anemia and elevated FGF23 are each highly associated with poor outcomes in patients with CKD, including increased odds for death and severe morbidity. Our goal was to test the correction of anemia and iron utilization on FGF23 production and its impact on mineral metabolism. Collectively, our data using two distinct therapeutic agents, namely EPO and FG‐4592, showed that both extended and acute reversal of anemia‐related manifestations in a model of CKD, respectively, were associated with a marked lowering of iFGF23. Consistent with known patient outcomes (Drueke et al., 2006; Pfeffer et al., 2009), an extended treatment with EPO increased hemoglobin levels in both CKD and control mice. Although our acute treatment protocol with FG‐4592 did not rescue hemoglobin levels or red blood cell production in the mice with CKD, this agent stimulated endogenous EPO production. Indeed, we did observe significantly elevated plasma EPO in control and CKD mice treated with FG‐4592, leading to increased bone marrow expression of Erythroferrone (Erfe). We also observed reduction of liver Bmp‐6, hepcidin, and ferritin mRNAs, which were consistent with EPO‐mediated Erfe production. Increased serum phosphate is associated with significant detrimental effects on CKD patients, including worsening of vascular calcifications leading to cardiac disease (Adeney et al., 2009; McGovern et al., 2013). Importantly, in the mice with CKD and treated with EPO or FG‐4592, despite the > 70% reduction in iFGF23 blood phosphate did not increase, and a restoration of kidney 1,25D metabolizing enzymes was observed. The difference in iFGF23 from peak elevated levels to the reduced concentrations following EPO and FG‐4592 treatments could reflect the “iron/anemia sensitive” portion of iFGF23 production during CKD. Despite this marked lowering, circulating iFGF23 concentrations remained well above normal physiological levels. Therefore this remaining proportion of iFGF23 could collectively reflect additional stimulators of FGF23 in CKD, such as hyperphosphatemia, inflammation, and PTH among others.

Strengths of this work included using the adenine model of CKD that displayed associated anemia as well as the known human phosphate‐related phenotypes. This model responded similarly to clinical therapeutics, including direct EPO injection and HIF‐PHDi, which completely rescued the prevailing anemia and acutely improved markers of iron handling, respectively. In this regard, with EPO treatment, red blood cell indices were returned to normal ranges. Whereas patients with CKD receiving EPO can have low serum iron requiring supplementation, it is possible herein that the elevated serum iron we observed in the CKD‐EPO group was potentially due to inhibiting further erythropoiesis and limiting iron utilization since a polycythemic state was reached. Another possibility is there could be stimulation of Erfe, leading to decreased hepcidin and increased iron absorption. In addition, FG‐4592 treatment markedly induced serum EPO and corrected liver iron utilization markers in the CKD group. Although it is not clear whether the increase in serum iron or the combination of elevated iron and reversal of anemia suppressed FGF23 in our study, it is likely that a major portion of the elevated FGF23 observed in the CKD mice could be attributed to defects in iron homeostasis. In a recent report, iron‐deficient mice with CKD receiving acute high dose EPO had no changes in iFGF23 after 6 and 24 hr, whereas iFGF23 was increased in normal, iron replete mice during the same timeframe (Hanudel, Eisenga, et al., 2018). Thus, the use of ESAs in the treatment of CKD may provide benefit in terms of mineral metabolism, as previous work using iron injections alone in a rat model of CKD did not have major effects on plasma FGF23 levels (Gravesen, Hofman‐Bang, Mace, Lewin, & Olgaard, 2013). Although the aforementioned short‐term EPO and FG‐4592 in vitro and in vivo studies caused FGF23 increases in normal animals and cells, the reduction of circulating FGF23 by improving iron utilization in CKD supports that suppression of FGF23 over the long term is a stronger effect than the acute stimulatory ability of ESAs on FGF23 synthesis. Therefore, a potential ESA off‐target effect of stimulating FGF23 production could be hypothesized. However, as a class these drugs may instead reduce FGF23 and provide ancillary patient benefit, although long‐term prospective trials are needed to test this hypothesis.

Limitations of our studies included the fact that FG‐4592 delivery was relatively short term. This approach was undertaken to assure that the mice with CKD would have sufficient residual kidney function to produce EPO in response to this analog, in line with previous studies of HIF‐PHDi. We found that the lower serum iFGF23 observed after FG‐4592 treatment was coincident with reduced liver ferritin and hepcidin mRNAs, as well as low serum iron. These results support the concept that even at very early stages of improving systemic iron utilization, FGF23 transcription, and cellular processing are sensitive to iron adaptation. Future work could focus upon performing longer term studies with EPO and HIF‐PHDi to identify whether FGF23 suppression is indirect through tissue–tissue interactions, or whether iron/oxygen content directly reduces anemia‐driven FGF23 production via suppression of HIF1‐2α transcriptional activity in osteocytes. Additionally, this study was not designed to fully distinguish the effects of reduced iFGF23 levels in the context of increased EPO, therefore, conditional‐deletion approaches could define EPO‐ and FGF23‐specific targets. In summary, our work demonstrated that the major portion of elevated iFGF23 in CKD is likely due to the associated anemia/iron‐deficiency, is therapeutically responsive to the control of iron utilization, and may thus provide modifiable patient benefit for mineral handling during CKD.

## CONFLICT OF INTEREST

KEW receives royalties from Kyowa‐Hakko‐Kirin Pharmaceutics, Inc. The other authors have no conflicts.

## AUTHOR’S CONTRIBUTION

MLN, ELC, MRA, and KEW contributed to the study design. MLN, ELC, PN, EAS, SPT, and RA collected and analyzed data. MLN, ELC, MRA, and KEW wrote, and critically revised the final draft of the manuscript.
